# Levels of Organochlorine Pesticides and Heavy Metals in Surface Waters of Konya Closed Basin, Turkey

**DOI:** 10.1155/2013/849716

**Published:** 2013-02-20

**Authors:** Mehmet Emin Aydin, Senar Ozcan, Fatma Beduk, Ali Tor

**Affiliations:** Department of Environmental Engineering, Necmettin Erbakan University, 42060 Konya, Turkey

## Abstract

The concentrations of organochlorine pesticides (OCPs), including **α**-, **β**-, **γ**-, and **δ**-hexachlorocyclohexane (HCH), heptachlor, heptachlor epoxide, dieldrin, aldrin, endrin, endrin aldehyde, endrin ketone, endosulfan I, endosulfan II, endosulfan sulfate, p,p′-DDE, p,p′-DDD, p,p′-DDT, methoxychlor, chlordane I, chlordane II, and heavy metals, such as As, Cr, Cu, Fe, Mn, and Ni in surface water samples from the Konya closed basin were determined to evaluate the level of contamination. Among all HCH isomers, **β**-HCH is the main isomer with a concentration range of 0.015–0.065 **μ**g/L. DDE, DDD, and DDT were almost determined in all samples, in which DDE isomer had the highest concentration ranged from not detected to 0.037 **μ**g/L. In all studied OCPs, aldrin showed the highest concentration at 0.220 **μ**g/L. The concentrations of heavy metals in water samples were observed with order: Mn < Cu < Ni < As < Cr < Fe. In some samples, As, Fe, and Cr concentrations exceeded the drinking water quality recommended by EU, US EPA, WHO, and Turkish Regulation, while Cu, Ni, and Mn concentrations are below the guideline values. The levels of both OCPs and heavy metals were also compared with other previously published data.

## 1. Introduction

Heavy metals occur naturally in soils and rocks entering the biogeochemical cycle. Anthropogenic releases, including industrial and domestic effluents, urban storm, water runoff, landfill leachate, atmospheric sources, coal-burning power plants, nonferrous metal smelteries, iron and steel plants, and dumping of sewage sludge can give rise to higher concentrations of the metals relative to the normal background values [1–3]. Heavy metals that enter aquatic system are ultimately deposited in suspended particulate matter and sediments that can be a long-term source of contamination [4]. Excess of some essential metals can damage human health, and nonessential metals can be toxic at even very low concentrations [5, 6].

Organochlorine pesticides (OCPs) have been used for decades in control of agricultural pests. Although most of OCPs were banned in many developed countries, some developing countries are still applying them for agricultural and public health purposes due to their low cost [7]. They accumulate in the environment owing to their persistence and lipophilic properties [8, 9]. High levels of OCPs are still being detected in various environmental media, especially in seafood [10].

Konya watershed is a closed basin and has 4.52 billion m^3^ water capacity. Konya closed basin has semiarid climate and limited water sources. Water shortage is faced because of increasing water demand due to the increasing population and expansion of the agricultural and industrial production. Uncontrolled drilled bore-holes for irrigation purposes and water abstraction intensified water shortage. Determination of the quality of existing limited water sources in Konya closed basin and identifying pollutant sources is of great importance. Most of the scientific efforts on the basin were carried out for extensive exploitation of groundwater that causes sinkhole development. On the other hand, a monitoring of the organic and inorganic contamination of water sources of the basin is lacking. Detection and quantification of a low contamination of OCPs and heavy metals in waters have become vital due to the toxicity of these contaminants to living organisms and humans [11]. Adverse effects of these contaminants on human health make it necessary to determine the concentration of these elements in water sources. Therefore, the objectives of this study are to investigate the levels of heavy metal (As, Cr, Cu, Fe, Mn, and Ni) and OCPs, including *α*-, *β*-, *γ*-, and *δ*-hexachlorocyclohexane (HCH), heptachlor, heptachlor epoxide, dieldrin, aldrin, endrin, endrin aldehyde, endrin ketone, endosulfan I, endosulfan II, endosulfan sulfate, p, p′-DDE, p, p′-DDD, p, p′-DDT, methoxychlor, chlordane I, and chlordane II in selected surface waters that can be drinking water sources in Konya closed basin, in order to provide a sufficient data for developing realistic risk reduction measures.

## 2. **Experimental**

### 2.1. Reagents and Solvents

All chemicals used were of analytical grade. The mixed standard of OCPs, including *α*-, *β*-, *γ*-, and *δ*-hexachlorocyclohexane (HCH), heptachlor, heptachlor epoxide, dieldrin, aldrin, endrin, endrin aldehyde, endrin ketone, endosulfan I, endosulfan II, endosulfan sulfate p, p^1^-DDE, p, p^1^-DDD, p, p^1^-DDT, methoxychlor, chlordane I, and chlordane II was obtained from Accustandard Co. (NewHaven, CT, USA). Standard stock solutions of 10 mg/L were prepared in methanol for each organic group.

Acetone, *n*-hexane, and dichloromethane were of residue grade (Merck, Germany). Anhydrous sodium sulphate was obtained from Merck, Germany. Metal standard stock solutions including As, Cu, Cr, Fe, Mn, and Ni were also from Merck. All solutions were stored in the dark at 4°C. 

### 2.2. Study Site

Konya closed basin is the largest closed basin of Turkey. There are many streams, lakes, and wetland areas. The water sources of Konya closed basin are only rainfall. After completing the circulation from underground and from surface in the basin, the water reaches to Salt Lake. Due to its large grassy steppes, biological diversity, and wetlands, Salt Lake is one of the 200 most important ecological regions in the world [12]. Konya closed basin, especially in recent years, faced several negative effects. Lack of rainfall and water sources, climate change and drought, development of industry, and untreated domestic and industrial wastewater discharges, nonefficient water consumption for agricultural purposes, drainage water from agriculture, drop in groundwater level, solid waste disposal problem are main sources of these effects. 

**Figure 1:**
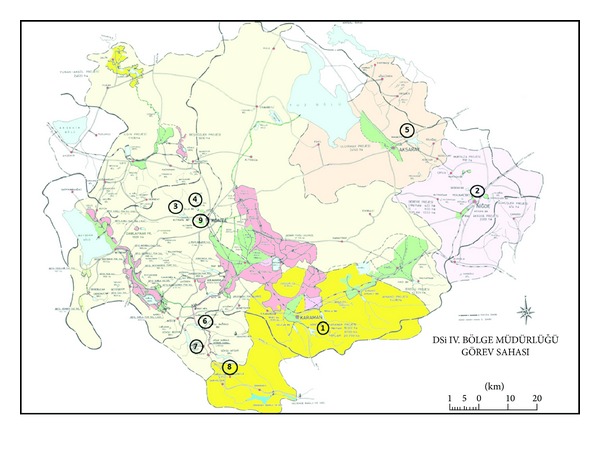
Map of the study area and sampling points (1) Ibrala stream; (2) Kirkgozler spring; (3) Basarakavak stream; (4) Meram stream; (5) Mamasin dam; (6) Bagbasi dam derivation tunnel inlet; (7) Bozkir stream; (8) AfsarIlicapinar stream; (9) Altinapa dam).

In this work, 9 monitoring stations were selected for investigation of OCPs and heavy metal pollution within the basin. All sampling locations are marked on the site map of the study area (Figure 1). 

### 2.3. Analytical Methods

The sampling, preservation, transportation, and analysis of the water samples were performed following standard methods. All samples were collected free of air bubbles in 1 L, glass containers and they were stored in the dark at 4°C. Water samples collected from sampling points were analyzed for heavy metals (As, Cu, Cr, Fe, Mn, and Ni) and OCPs. Concentrations of heavy metals in water samples were determined by using Inductively Coupled Plasma/Mass Spectrometry (ICP/MS, Perkin Elmer) following standard guidelines and procedures [13]. Analytical curves were drawn using eight points. The calibration curves gave a high level of linearity for all studied heavy metals with correlation coefficients ranging between 0.9983 and 0.9999. The LOD values were found to be in the low ppb level, ranging from 0.0047 to 0.2210 *μ*g/L.

In order to determine trace levels of OCPs, an extraction and preconcentration step is necessary. LLE procedure was adopted from US EPA Method 3510C [14]. Water sample (200 mL) was placed in a 250 mL separatory funnel. The extraction was carried out three times with 20 mL  of dichloromethane. The extracts were combined and dried with anhydrous sodium sulfate. The resulting extract was concentrated to 1 mL  using rotary evaporator (Buchi B-160 Vocabox, Flawil 1, Switzerland) and gentle nitrogen stream and transferred into the vial. Then, gas chromatography mass spectrometer (GC/MS) analysis was performed as described in Section 2.4. The limits of detection (LODs), based on a signal-to-noise ratio (S/N) of 3 [15] for OCPs, were in the range of 0.005–0.052 *μ*g/L for OCPs. The calibration techniques for OCPs are the external standard multipoint calibration using at least five concentration levels. For spiking concentration of OCPs at levels of 0.1 *μ*g/L, 1 *μ*g/L, and 5 *μ*g/L, the recovery rates after extraction and concentration procedures were in the range of 85–102% with relative standard deviation <5%.

### 2.4. Gas Chromatography/Mass Spectrometry (GC/MS) Conditions

The determination of OCPs was carried out by gas chromatograph (GC, Agilent 6890N, Agilent Technologies, Palo Alto, CA, USA) equipped with mass-selective detector (MS, Agilent 5973, Agilent Technologies, Foster City, CA, USA). The features and operating conditions of GC/MS system were as follows: GC, equipped with programmed temperature vaporizing (PTV) injector, DB-5 MS 5% phenylmethylsiloxane fused silica capillary column (30 m  length, 0.25 m  i.d. and 0.25 *μ*m film thickness), and helium (purity 99.999%) as carrier gas at constant flow-rate of 1.9 mL/min. The injection volume was 2 *μ*L and PTV was operated in splitless mode. Injections were performed by an Agilent 7683 B Series automatic injector (Agilent Technologies, Palo Alto, CA, USA). PTV program was as follows: 80°C, 12°C/s to 350°C and hold at 350 °C for 2 min. The temperature of the ion source and MS transfer line were maintained at 170°C and 280°C, respectively. The oven program for OCPs was the same as reported by Ozcan [16]. MS detector was operated in selected ion monitoring (SIM) mode.

## 3. Results and Discussion

### 3.1. Levels of OCP Residues in Water Samples

The concentration ranges of OCPs residues and total concentrations of HCHs (*∑*HCHs) and DDTs (*∑*DDTs) in surface water samples are presented in Table 1. All HCH isomers were determined in water samples from Konya closed basin. For March 2012 and August 2012, the concentrations of *∑*HCHs were in the range (0.015–0.065) *μ*g/L and (not detected-0.025) *μ*g/L, respectively. In addition, the levels of each HCH isomer determined in the samples taken from March 2012 were higher than those taken from August 2012. For sampling period March 2012, of all HCH isomers investigated, *β*-HCH was the dominant substance determined with a range of 0.005–0.020 *μ*g/L. Qu et al. [17] reported that the commercial HCH consists of a mixture of HCH isomers including 65–70% of *α*-HCH, 7–10% of *β*-HCH, 14-15% *γ*-HCH, and 10% of *δ*-HCH and other constituents. Because *α*-HCH is instable in the environment, it is easy to metabolized into *β*-HCH [17]. Thus, *β*-HCH is the main isomer determined in most water samples. 

Comparison of HCH levels in the water samples of this study with surface water samples from different region in Turkey, the range of *∑*HCHs (0.015–0.065 and not detected-0.025 *μ*g/L for each sampling period) was less than those found water sample from Küçük Menderes River (0.101–0.559 *μ*g/L) [18]. In another study of Turgut and Fomin [19], the concentration of *δ*-HCH was 2.065 *μ*g/L, which is more than levels of *δ*-HCH determined in our study.

In the case of DDT isomers, DDE, DDD, and DDT were determined in all water samples. Among these isomers, DDE was detected with the highest concentration range between not detected to 0.037 *μ*g/L. This result is most probably the result of banning the use of DDT in Turkey. The range of ***∑***DDTs' levels in this study was considerably lower in comparison to another river and water reservoir in Turkey [19–21] and in Bay of Ohuira, Mexico [22].

The level of heptachlor in the samples taken from March 2012 was in the range from not detected to 0.005 *μ*g/L and it was not determined in the samples from August 2012. The concentration of heptachlor in this study was more than the sample (5.9 pg /L) of Lake Baikal, Siberia, which is reported by Kuckllck et al. [23]. 

The concentration range of aldrin in all samples taken from March (0.055–0.220 *μ*g/L) and August 2012 (0.005–0.040 *μ*g/L) was more than those from dieldrin (not detected-0.015 *μ*g/L and not detected-0.010 *μ*g/L for March and August 2012, resp.). According to Blus et al. [24], aldrin is rapidly broken down to dieldrin. Based on this result, it can be concluded that fresh input of aldrin in the Konya closed basin still occurs.

Among the endosulfans, the highest concentration range belongs to endosulfan-II (0.095–0.165 *μ*g/L and 0.005–0.080 *μ*g/L for March and August 2012, resp.). This result was against the results which is reported by Osuna-Flores and Riva [22] that the endosulfan-I had the highest concentration (0.010–1.260 *μ*g/L) in surface water samples from Bay of Ohuria, Mexico. Turgut [18] also reported that endosulfan sulfate had the highest concentration with the range of 0.028–0.123 *μ*g/L in the samples of Küçük Menderes River in Turkey. 

The endrin concentration in this study ranged from 0.005 to 0.015 *μ*g/L. Other endrin metabolites (endrin aldehyde and endrin ketone) were also determined in samples of March and August 2012 (Table 1). The level of endrin was comparable with that reported for the water samples of Küçük Menderes River [18] and it was less than those in the river and water reservoir in Göksu Delta with a maximum median concentration of 21 *μ*g/L [20].

### 3.2. Levels of Heavy Metals in Water Samples

The results on the levels of heavy metals in surface waters in Konya closed basin are shown in Table 2. In March 2012 among the heavy metals, Cr had the least concentration range (not detected-5.49, median: 3.14 *μ*g/L). While, the results for samples of August 2012 showed that Cu had the least concentration range (0.60–5.63, median: 1.10 *μ*g/L). It is seen in Table 2 that the order of the median level of heavy metals in water samples from March and August 2012 was Mn < Cu < Ni < As < Cr < Fe. 

The limit values of heavy metals for drinking water quality recommended by EU [31], US EPA [32], WHO [33] and Turkish Regulation [34] and for the protection of freshwater aquatic life [35] are presented in Table 3. A comparison for the maximum concentrations of As, Cr, Cu, Fe, Mn and Ni in surface waters of the Konya closed basin with those in other rivers is also presented in Table 4. 

The concentrations of As in some water samples (27% of all samples) exceeded the maximum permitted concentration for drinking water quality guidelines. This poses a threat to human health because of high carcinogenic effect of As [36]. Similarly, Fe concentration of 22% of all samples were higher than the guideline values. The concentration of Cr was lower than the limits values, except for US EPA limit. Cu, Ni, and Mn had concentrations that are below the guideline values. High level of Fe may be a source of high As concentration in the samples. Reduction of iron oxyhydroxide (FeOOH) and release of its sorbed As load to solution which is the most common mechanism that pollutes water [37].

## 4. Conclusion

The results from this study provide important data for the literature because it is the first study that report the levels of OCPs and heavy metals of surface water in Konya closed basin (Turkey). The results showed the most of studied OCPs were determined in water samples. In general, the concentrations of OCPs were mostly comparable or less than data in the literature. Among the heavy metals, As and Fe were of concentrations that are higher than EU, WHO, US EPA, and Turkish regulation limits for drinking water. However, Cu, Ni, and Mn had lower concentrations than regulation limits. Arsenic is a threat to human health with its high carcinogenic effect. Chronic effects on humans may be caused by prolonged consumption of water with even very low concentrations. The high level of Fe was observed in water sources, which is a possible source of As enrichment in the study area. It is concluded that a risk determination about the occurrence and source of As and Fe is needed for the whole basin and realistic risk reduction measures must be developed.

## Figures and Tables

**Table 1:** The levels of OCPs residues in surface water samples in March 2012 and August 2012 (*µ*g/L).

OCPs	March 2012	August 2012
	Minimum–maximum	Minimum–maximum
*α*-HCH	0.005–0.010	nd–0.005
*β*-HCH	0.005–0.020	nd–0.005
*γ*-HCH	0.005–0.010	nd–0.005
*δ*-HCH	nd–0.015	nd–0.010
***∑*HCHs**	**0.015**–**0.065**	**nd**–**0.025**
p,p′-DDE	nd–0.037	nd–0.010
p,p′-DDD	nd–0.005	nd
p,p′-DDT	nd–0.005	nd
***∑*DDTs**	**nd**–**0.047**	**nd**–**0.010**
Heptachlor	nd–0.005	nd
Heptachlor epoxide	nd–0.025	0.005–0.015
Aldrin	0.055–0.220	0.005–0.040
Chlordane-I	nd–0.005	nd
Chlordane-II	nd	nd–0.010
Endosulfan-I	0.005–0.025	nd–0.005
Endosulfan-II	0.095–0.165	0.005–0.080
Endosulfan sulfate	nd–0.015	nd
Dieldrin	nd–0.015	nd–0.010
Endrin	0.005–0.015	0.005–0.010
Endrin aldehyde	0.015–0.035	nd–0.005
Endrin ketone	nd–0.010	nd–0.010
Methoxychlor	nd	nd

nd: not detected.

**Table 2:** The levels of heavy metals in surface water samples in March 2012 and August 2012 (*µ*g/L).

Heavy metals	March 2012	August 2012
	Minimum–maximum (median)	Minimum–maximum (median)
As	nd–14.77 (3.11)	1.32–91.11 (4.73)
Cu	nd–7.89 (1.85)	0.60–5.63 (1.10)
Cr	nd–5.49 (3.14)	13.37–40.94 (19.73)
Ni	nd–15.46 (2.48)	1.24–6.38 (2.30)
Fe	nd–1273 (266)	148.89–296.60 (165.53)
Mn	nd–39.22 (1.19)	0.04–8.43 (0.29)

nd: not detected.

**Table 3:** Limit values for heavy metals for drinking water quality and protection of freshwater aquatic life, *μ*g/L.

Heavy metals	TRWHC	WHO	USEPA	EU
As	10	10	10	10
Cr	50	50	10	50
Cu	50	50	10	50
Fe	200	300	300	200
Mn	50	500	50	50
Ni	20	—	—	20
Zn	—	—	5000	—

**Table 4:** The maximum concentration of As, Cr, Cu, Fe, Mn, and Ni in water samples from other rivers in the literature.

Location	Maximum concentration, *µ*g/L						
	As	Cr	Cu	Fe	Mn	Ni	Reference
Khoshk River, Iran	—	550	60	—	540	200	[25]
San Petro River, Mexico	160	212	200	1800	4620	300	[26]
Sakarya River, Turkey	—	228	4362	—	—	4977	[27]
Axios River, Greece	10.9	4.2	13.7	—	—	8.2	[28]
Odra River, Poland	8.1	12.7	54.6	1861	353	27.2	[29]
Vardar River, Macedonia	—	—	98	1904	249	—	[30]
Samples in Konya closed basin	91.11	40.94	7.89	1273	39.22	15.46	This study
